# Knowledge of first aid methods and attitude about snake bite among medical students: a cross sectional observational study

**DOI:** 10.1186/s12995-018-0210-0

**Published:** 2018-08-15

**Authors:** Nuwadatta Subedi, Ishwari Sharma Paudel, Ajay Khadka, Umesh Shrestha, Vipul Bhusan Mallik, K. C. Ankur

**Affiliations:** 1 Department of Forensic Medicine, Gandaki Medical College, Pokhara, 33700 Nepal; 2 Department of Community Medicine, Gandaki Medical College, Pokhara, Nepal; 3 Gandaki Medical College, Pokhara, Nepal

**Keywords:** First aid, Medical students, Public health, Snake bite

## Abstract

**Background:**

Snake bite is a neglected public health problem in tropical and subtropical region. The study was conducted with objectives to determine the knowledge of first aid methods in snake bite and the perception of snake bite among the medical students of Gandaki Medical College, Pokhara, Nepal.

**Methods:**

We conducted a cross sectional survey among 302 (231 preclinical and 71 clinical) Bachelor of Medicine and Bachelor of Surgery (MBBS) students of Gandaki Medical College using a pretested questionnaire to assess the knowledge of first aid of snake bite based on WHO protocol and perception of snakebite. The study duration was from January to May 2018. The total score of the knowledge was obtained and compared among variables using Mann-Whitney U test. Chi square test was used for comparing the responses with the level of students. *P* value of < 0.05 was considered as significant.

**Results:**

Among 302 respondents, 193(63.9%) were from Mountain districts. The families of 25 (8.3%) respondents were bitten by snakes. The correct responses were significantly higher from the 71 (23.5%) clinical students for most of the questions and the knowledge score of clinical students was significantly higher than the 231 (76.5%) preclinical students. Twenty eight (9.27%) students believed that the snake should be killed after it bites the victim and 25 (8.28%) believed that the snake will capture the image of the offender who teases it and takes revenge later. School books were the commonest source of such knowledge among the preclinical students.

**Conclusion:**

Most of the preclinical students had inadequate knowledge of first aid of snake bite. The common source of the knowledge was school books which often provide faulty knowledge. Only a few students had negative perception about snakes. Incorporation of proper first aid measures in the textbooks of various levels is essential.

## Background

Snakebite is a common and neglected public health problem in tropical and subtropical region affecting people mostly of lower socioeconomic group. It mostly affects the farmers and those who work in the fields and thus one of the occupational injury. The public health issues of snakebite is neglected globally [1], and it has only been added to WHO’s list of neglected tropical diseases in June 2017. The annual incidence of snake bites in Nepal is 15,000 and 10% of them are with envenomation with 10% mortality rate among the bite by poisonous snakes. There might be under reporting of the snakebite cases from Nepal and in the latest time, the actions have been taken by increasing supervisory visit to the reporting sites to overcome it [2].

The public of Nepal have many misconceptions about snakes and snake bite, that can deleteriously affect the treatment and its prognosis [3]. The patients of snake bite often seek traditional healers [4] and get faulty first aid measures before presenting to the hospital as reported from Nepal [3, 5–8] India [8–11], and even in China [12]. Improper first aid in snake bites causes more harm rather than improvement [13]. The textbooks mentioning the first aid measures of snake bite published from Nepal advocate up to 100% different than the published guidelines, omitting the indicated ones and recommending faulty and deleterious methods [14, 15]. This can lead to deep rooted false knowledge about first aid of snake bite to the people, deleteriously affecting the prognosis of the snake bitten victims if such techniques are used. This study can be useful to determine the existing knowledge among the MBBS students of Gandaki Medical College who are the future health care providers.

## Methods

### Objectives of the study

The study was conducted with objectives to determine the knowledge of first aid methods in snake bite and the perception of snake bite among the medical students of Gandaki Medical College.

### Study design and population

It is a cross sectional study conducted among the MBBS students of Gandaki Medical College, Pokhara from January to May 2018. All the students present at the time of collection of the questionnaire and consenting for participation were included in the study. The students were approached by the authors after the end of their regular class and explained about the objectives of the study and the way to answer the questionnaire. They were encouraged to fill up the questionnaire to the best of their knowledge and perception. They were also informed that the responses would be confidential, and no any identifiers were used in the forms while filling up the questions so that the students were encouraged to answer confidently.

We had chosen the MBBS students as most of the talented students are enrolled in medical education in our country and their responses about the knowledge of snake bite would reflect the highest possible correct responses from the students if other streams are considered. They are also exposed to the formal education of snake bite treatment, the types of snake and the myths and truth about snakes during the third year MBBS in Forensic medicine and Toxicology. So, we could compare the knowledge of students before and after the such formal education.

A questionnaire including the participants’ knowledge about the first aid methods for snake bite was prepared based on the WHO protocol of management of snake bite [16]. The sex, district of upbringing and information whether their immediate family members were bitten by snakes were recorded. Two questions were added to assess the perception of the students about snakes and snake bite. We also included a question asking the respondents about the source of the knowledge that they had acquired. The questionnaire was pretested among 33 MBBS students of another medical college who were not included in the main study. The discrepancies were corrected, and the final questionnaire was administered to the participants.

At the college, the students are categorised as clinical from third year MBBS. But as the students were just admitted to the third year and had not got the formal clinical education, moreover in the snake bite management and first aid protocols, they were included among the preclinical students in our analysis.

The responses for the knowledge-based questions were “true”, “false” and “do not know”. For the questions assessing the knowledge of first aid of snake bite and its management issues, a score of one was given for each correct response and zero for the incorrect response and for “do not know” response. The total score was calculated with a minimum of zero and maximum nine. The overall knowledge was considered inadequate if the score was less than 70% (score of six and less) and adequate if more than 70% (score of seven and more) as done by Michael et al. [17].

### Statistics

The data was collected and entered in MS Excel and further analysis done using SPSS version 16.0. To check whether the data was normally distributed, Kolmogorov Smirnov test was used. Non-parametric tests were used for the not normally distributed data. The level of knowledge was compared among sex, level of students, region of upbringing and whether their family members were bitten by snakes using Mann-Whitney U test. Chi square test was used for comparing the responses with the level of students (Preclinical and clinical) *P* value of < 0.05 was considered as significant.

### Ethical consideration

Consent was obtained from the study participants before administering the questionnaire. We had adhered to the Declaration of Helsinki in enrolling our participants. The study was ethically approved by the Institutional Review Committee of Gandaki Medical College before commencing the study.

## Results

There were a total of 395 MBBS students at Gandaki Medical College during the study period among which 302 (76.46%) were present at the time of data collection. Among 302 questionnaires distributed to the students, all of them were filled and returned. (Response rate, 100%). Among a total of 302 MBBS students participating in the study, 153 (50.7%) were males and 149 (49.3%) were females. The students with formal exposure to snake bite management were categorized as clinical students while those without the exposure as preclinical students. There were 71 (23.5%) clinical students participating in the study. A total of 193(63.9%) of the students were from Mountain districts and 109 (36.1%) from Terai districts. Most of the students; 275 (91.1%) were educated from private schools and 27 (8.9%) from government schools up to secondary education. The families of 25 (8.3%) respondents were bitten by snakes. (Table 1).

**Table 1:** Demographics of the participants

Variables	Number	Percent
Sex		
Males	153	50.7
Females	149	49.3
Level		
Preclinical	231	76.5
Clinical	71	23.5
School up to secondary level		
Government	27	8.9
Private	275	91.1
Region of upbringing		
Mountain	193	63.9
Terai	109	36.1
Family members bitten by snake		
Yes	25	8.3
No	277	91.7

The responses of the students for the knowledge questions are shown in Table 2. A total of 235(77.8%) responded that tight bands (tourniquets) should be applied around the limb proximal to the bite site, which is not correct. One hundred and seventy (56.29%) students were aware of pressure immobilization bandages to be applied around the bite site, 299 (99.01%) responded that the snake bite patient should be transported to the hospital soon and 261 (86.42%) knew that envenomation be cured by anti-venom therapy. When the knowledge of the students was categorized as adequate (Score more than 70%, or seven and more) and inadequate, it was explored that 29 out of 231 (12.6%) preclinical and 49 out of 71 (69%) clinical students had adequate knowledge about first aid of snake bite.

**Table 2:** Respondents’ knowledge of first aid of snake bite

Variables	Yes n (%)	No n (%)	I don’t know n (%)
Should local incisions or pricks/punctures be made over the bite site?	99 (32.78)	142 (47.02)^a^	61 (20.12)
Should healthy volunteer suck the venom out of the wound?	86 (28.48)	204 (67.55)^a^	12 (3.97)
Should tight bands (tourniquets) be applied around the limb proximal to the bite site?	235 (77.81)	59 (19.54)^a^	8 (2.65)
Should pressure immobilization bandages be applied around the bite site?	170 (56.29) ^a^	47 (15.56)	85 (28.15)
Is electric at the site of bite useful?	13 (4.30)	215 (71.19)^a^	74 (24.50)
Is topical instillation or application of herbs beneficial?	115 (38.08)	83 (27.48)^a^	104 (34.44)
Should the snakebite patient be transported to the hospital soon after the bite?	299 (99.01)^a^	2 (0.66)	1 (0.33)
Can envenomation be cured by anti-venom therapy?	261 (86.42)^a^	1 (0.33)	40 (13.24)
Are all snake bites associated with envenomation?	56 (18.54)	176 (48.34)^a^	70 (23.18)

^a^Denotes correct responses

The knowledge of first aid and treatment of snake bite categorized as current and incorrect responses and comparison among the level of students is shown in Table 3. The correct responses were significantly higher from the clinical students for most of the questions except for transportation of the snakebite patient to the hospital as soon as possible. (χ^2^ = 0.931, *p* = 0.446, df = 1).

**Table 3:** Knowledge categorized as current and incorrect responses and comparison among the level of students

SN	Knowledge on snake bite and First Aid and treatment Measures	Correct Response n (%)	Incorrect response n (%)	χ2	*P* Value
1	Should local incisions or pricks/punctures be made over the bite site?				
	Preclinical	81 (35.07)	150 (64.93)	56.372	< 0.001
	Clinical	61 (85.92)	10 (14.08)		
2	Should healthy volunteer suck the venom out of the wound?				
	Preclinical	137 (59.31)	94 (40.69)	25.490	< 0.001
	Clinical	65 (91.55)	6 (8.45)		
3	Should tight bands (tourniquets) be applied around the limb proximal to the bite site?				
	Preclinical	19 (8.22)	212 (91.78)	79.097	< 0.001
	Clinical	40 (56.34)	31 (43.66)		
4	Should pressure immobilization bandages be applied around the bite site?				
	Preclinical	118 (51.08)	113 (48.92)	10.836	0.001
	Clinical	52 (73.24)	19 (26.76)		
5	Is electric current at the site of bite useful?				
	Preclinical	155 (67.10)	76 (32.90)	8.024	0.005
	Clinical	60 (84.51)	11 (15.49)		
6	Is topical instillation or application of herbs beneficial?				
	Preclinical	54 (23.38)	177 (76.62)	8.315	0.004
	Clinical	29 (40.85)	42 (59.15)		
7	Should the snakebite patient be transported to the hospital soon after the bite?				
	Preclinical	228 (98.70)	3 (1.30)	0.931	0.446^a^
	Clinical	71 (100.00)	0 (0.00)		
8	Can envenomation be cured by anti-venom therapy?				
	Preclinical	191 (82.68)	40 (17.31)	11.713	0.001
	Clinical	70 (98.59)	1 (1.410		
9	Are all snake bites associated with envenomation?				
	Preclinical	115 (49.78)	116 (50.22)	29.159	< 0.001
	Clinical	61 (85.92)	10 (14.08)		

^a^Fisher Exact test

A total of 29 (12.6%) preclinical and 49 (69%) clinical students had adequate knowledge (score of seven and more) about the knowledge of snake bite. The total score of knowledge was not normally distributed as analyzed by Kolmogorov Smirnov test (*p* < 0.001, df = 302). The median score was five, minimum two and maximum nine. The comparison of the score of knowledge among the variables using Mann-Whitney U test is shown in Table 4. The knowledge score of clinical students was significantly higher (U = 2015.5, *p* < 0.001) than the preclinical students. The knowledge was not significantly associated with sex (U = 10,039, *p* = 0.69), region of upbringing (U = 9633, *p* = 0.218), school up to secondary level (U = 3299, *p* = 0.333), and family members bitten by snake or not (U = 41,232, *p* = 0.075).

**Table 4:** Comparison of knowledge score among the variables

Variables	N	Rank average	Sum of ranks	U	Z	*p*-value
Sex						
Male	153	160.39	24,539.00	10039.00	−1.818	0.69
Female	149	142.38	21,214.00			
Level of students						
Preclinical	231	124.73	28,811.50	2015.50	−9.753	< 0.001
Clinical	71	238.61	16,941.50			
Region of upbringing						
Mountain	193	156.09	30,215.00	9633.00	−1.233	0.218
Terai	109	143.38	15,628.00			
School up to secondary level						
Private	275	153.00	42,076.00	3299.00	−0.969	0.333
Government	27	3677.00	3677.00			
Family member bitten by snake						
Yes	25	180.84	4521.00	41232.00	−1.780	0.075
No	277	148.85	41,232.00			

We had collected some responses about the perception of snakes which is displayed in Table 5. A total of 17 (7.35%) preclinical and 11 (15.49%) clinical students believed that the snake should be killed as far as possible after it bites the victim. Twenty (8.66%) preclinical and five (7.04%) clinical student believed that the snake will capture the image of the offender who teases it and takes revenge later.

**Table 5:** Perception of snake bite and snakes

SN	Perception of Snake bite	True n (%)	False n (%)	Do not know n (%)
1	The snake should be killed as far as possible after it bites the victim			
	Preclinical	17 (7.35)	201 (87.01)	13 (5.63)
	Clinical	11 (15.49)	59 (83.10)	1 (1.41)
2	The snake will capture the image of the offender who teases it and takes revenge later			
	Preclinical	20 (8.66)	183 (79.22)	28 (39.44)
	Clinical	5 (7.04)	57 (80.28)	9 (12.67)

We had asked the source of information of knowledge about snake bite to the preclinical students. The common sources were school books as responded by 127 (54.98%), television (122, 52.81%) and school teacher (88, 38.09%), newspapers (69, 29.87%), radio (52, 22.51%) and others (27, 11.69%).

## Discussion

The present study is a cross sectional questionnaire-based study conducted among the 302 first to final year MBBS students of Gandaki Medical College. The responses from the participants would reflect the knowledge and perception of snake bite of the knowledgeable graduate level students of Nepal. Moreover, as they undergo a formal training of the snake bite treatment, myths and misconceptions associated with snake bite, we could compare the knowledge of the students who had been formally trained and not.

Considerable number of students, mostly at the preclinical level still opt for obsolete and traditional means of first aid associated with snake bite. Some of the obsolete, traditional and deleterious measures still thought to be true first aid measures of snake bite are: making local incisions or pricks/punctures over the bite site, sucking the venom out of the wound by a healthy volunteer and tying tight bands (tourniquets) around the limb proximal to the bite site. These are performed with a belief that the venom would be taken out of the site of bite and prevent the spread of venom. Similar findings were presented in other studies [8–12, 18]. In a study among 39 patients with signs of snake bite envenomation in central Nepal, none of them had adopted WHO recommended first aid measures; pressure immobilization bandaging or local compression pad immobilization rather many of them had practiced traditional measures before getting admitted to the hospital [8]. More than half among 180 patients of snake bite had presented with application of tourniquet in an Indian study [19].

In a review about snake bite in South Asia, eight out of 15 studies showed that more than half of snake bite patients had used inappropriate and deleterious methods of first aid, among which tourniquets were used by a maximum number of patients [18]. The faulty and inappropriate first aid methods mentioned in the textbooks from primary to the bachelor’s level [14, 15] can be an important contributing factor that has caused such deep rooted faulty knowledge.

In contrast to our study, Sri Lankan farmers were against the faulty first aid methods like incising the bite site and application of tourniquets, though it was not practiced perfectly [20]. Even the medical practitioners of Nigeria [17, 21] did not have adequate knowledge of snake bite envenomation and only few doctors of Hong Kong were confident of snake bite treatment [22].

The only evidence based first aid technique of snake bite which delays the spread of venom is the pressure immobilization technique. The other first aid measures like tourniquet application are harmful and should be avoided [23]. The interference of the wound of snake bite would by incising, rubbing, vigorously cleaning, applying chemicals and herbs etc. can cause infection, increased absorption of the snake venom and increment of local bleeding. The use of tight (arterial) tourniquets, if applied around the proximal portions of the limb, these can cause severe pain as there will be gradual development of ischaemia on the limbs and can lead to gangrene of left in place for a long time [16].

The source of such knowledge could have passed on from generations. The main reason behind this faulty knowledge to the students could be due to the incomplete and inappropriate first aid methods mentioned in the textbooks of primary to university level. Such textbooks published from Nepal provide incorrect information up to 100% errors [14, 15]. It is very important to provide updated and accurate information in the textbooks so that the students will acquire proper information of first aid of snake bite and they can also disseminate the information to the public in society. The author NS had witnessed a television program from a National television where the outdated and deleterious first aid measures of snake bite were telecasted only a few years back. The concerned authorities should be very serious in this matter. The guideline of first aid of snake bite published by WHO recommends that at least no harm should be done to the victims of snake bite in an attempt to perform first aid measures in them [16]. The correct and updated information of first aid measures of snake bite has to be disseminated using newspapers, radio, television, internet etc.

The knowledge of the students about the first aid of snake bite was not significantly different in comparison with the variables like sex, type of school up to secondary level, district of upbringing and the status of family member bitten by snake. This indicates that the curriculum in private and public schools both of Nepal does not significantly include appropriate information which is supported by studies using textbooks from primary to university level [14, 15]. Almost all the students were positive about the transfer of the snakebite patients to the hospital soon after the bite. Most people know that definitive treatment of snake bite envenomation can be done at hospitals. Rapid transport of victims to a snake bite treatment center had decreased the mortality rate in a study from southeastern Nepal [3].

The knowledge of the clinical students was significantly higher than compared to the preclinical students. The third year MBBS curriculum of Forensic Medicine and Toxicology includes identification of snakes, management of snake bite including the established first aid methods, and myths and misconceptions associated with snakes and snake bite. The students are also practically demonstrated various types of poisonous and nonpoisonous snakes common in Nepal and South Asian region. The higher knowledge of the clinical students indicates that formal training can significantly correct the misconceptions about the deleterious first aid methods of snake bite. The potential medical personnel involved in the management of snake bite patients should be regularly updated of the advances and protocols. The increment in knowledge of snakes and competencies about the snake bite management by trainings and community education has also been demonstrated earlier [3, 4, 21].

Only 15% of all the 3000 species of snakes found globally are venomous [24] and the rest are non-venomous. When the snake bite is associated with envenomation, the definitive treatment is antivenom therapy and it should be administered when indicated [16]. The people have tendency to kill the snakes when they see them and more often when the snakes bite [25]. The snakes are important creatures to maintain ecological harmony and should preserved. In the attempt to kill the snakes, the people may be further bitten by it may be grievous. In our study, 28 (9.27%) respondents perceived that the snakes should be killed after it bites and 25 (8.28%) had the misconception that the snakes capture image of the offender who teases it and takes revenge later. The later could be one of the reasons that people would like to kill the snakes when they encounter them.

## Conclusion

Most of the preclinical students had inadequate knowledge of first aid of snake bite and many of them opted for obsolete and deleterious methods. The common source of the knowledge was school books which often provide faulty knowledge. Only a few students had negative perception about snakes. The students should be taught correct and updated methods of first aid of snake bite from the early stage of their education. If faulty technique is included in their curriculum, they might have deep rooted wrong knowledge which can ultimately affect the society.

## Abbreviations

MBBS: Bachelor of Medicine and Bachelor of Surgery
WHO: World Health Organization
